# Can the Neural Basis of Repression Be Studied in the MRI Scanner? New Insights from Two Free Association Paradigms

**DOI:** 10.1371/journal.pone.0062358

**Published:** 2013-04-30

**Authors:** Jo-Birger Schmeing, Aram Kehyayan, Henrik Kessler, Anne T. A. Do Lam, Juergen Fell, Anna-Christine Schmidt, Nikolai Axmacher

**Affiliations:** 1 Department of Epileptology, University of Bonn, Bonn, Germany; 2 Department of Medical Psychology, University of Bonn, Bonn, Germany; 3 German Center for Neurodegenerative Diseases (DZNE), Bonn, Germany; 4 Department of Medical Psychology, University of Ulm, Ulm, Germany; Inserm, France

## Abstract

**Background:**

The psychodynamic theory of repression suggests that experiences which are related to internal conflicts become unconscious. Previous attempts to investigate repression experimentally were based on voluntary, intentional suppression of stimulus material. Unconscious repression of conflict-related material is arguably due to different processes, but has never been studied with neuroimaging methods.

**Methods:**

We used functional magnetic resonance imaging (fMRI) in addition with skin conductance recordings during two free association paradigms to identify the neural mechanisms underlying forgetting of freely associated words according to repression theory.

**Results:**

In the first experiment, free association to subsequently forgotten words was accompanied by increases in skin conductance responses (SCRs) and reaction times (RTs), indicating autonomic arousal, and by activation of the anterior cingulate cortex. These findings are consistent with the hypothesis that these associations were repressed because they elicited internal conflicts. To test this idea more directly, we conducted a second experiment in which participants freely associated to conflict-related sentences. Indeed, these associations were more likely to be forgotten than associations to not conflict-related sentences and were accompanied by increases in SCRs and RTs. Furthermore, we observed enhanced activation of the anterior cingulate cortex and deactivation of hippocampus and parahippocampal cortex during association to conflict-related sentences.

**Conclusions:**

These two experiments demonstrate that high autonomic arousal during free association predicts subsequent memory failure, accompanied by increased activation of conflict-related and deactivation of memory-related brain regions. These results are consistent with the hypothesis that during repression, explicit memory systems are down-regulated by the anterior cingulate cortex.

## Introduction

Repression is a key concept of psychoanalytical theory. It suggests that contents which are related to internal conflicts are made unconscious [1]. Various psychodynamic therapies use this concept as a heuristic to explain psychological symptoms arising from repressed internal conflicts [2], [3]. Previous studies on the neural correlates of repression mostly used variants of the “directed forgetting” or “think/no-think” paradigms [4]–[6]. These studies describe increased activity in dorsolateral prefrontal cortex (DLPFC) and decreased activity in the medial temporal lobe during suppression of unwanted memories [4], [5]. Putatively, this corresponds to a top-down inhibition of declarative memory [7]–[9] by executive control [10]. These paradigms, however, are more closely related to voluntary memory suppression than to actual repression as operationalized in psychoanalytic theory, which is typically not conscious and related to an internal conflict [11], [12]. Results from research on emotion regulation [13] and hysterical conversion disorder [14] point to the role of cortical limbic structures such as medial prefrontal cortex and anterior cingulate cortex (ACC) in the unconscious modulation of activity in subcortical structures. The ACC is, among other functions, widely known for its pivotal role in the detection and processing of conflicts, including those involving emotional [15]–[19] and autobiographical material [20], [21]. These studies suggest that automatic regulatory processes rely on different brain structures than voluntary suppression [12], [22].

Another vein to study repression empirically started with C. G. Jung [23] and is based on the idea that the level of physiological arousal during free association indicates whether contents are related to repressed conflicts. In these paradigms, words are generated by free association – i.e., participants are presented a cue word and asked to name the first word which comes to their minds. The technique of free association is therapeutically used to overcome resistance towards revelation of repressed conflicts. According to psychodynamic theory, if a generated word is thematically related to an internal conflict, it would be subject to repression itself. Clinical experience suggests that repression of internal conflicts indeed leads to autonomic arousal [2], which can be measured by an increase in skin conductance responses (SCRs) and reaction times (RTs) [24]. Consistent with the idea that repression impairs conscious access, subsequent cued recall of these associations is impaired for words generated under high autonomic arousal [25]–[27]. Possibly, arousal in this paradigm could reflect a relation of the presented cue and/or the generated association to a previously repressed memory content. According to psychodynamic theory, the repressed content may be accessible by free association, which is thought to reduce censorship [28]. This, however, would then lead to arousal and to a repeated effort to repress the conflict-related contents, preventing subsequent conscious memory for the freely associated words (for more details, please see the section entitled “Interpretation issues” in the Discussion). Notably, this effect of arousal is opposite to the facilitating effect of arousal on memory formation [29]–[34] which is typically observed if stimuli are not self-generated by free association; in the Discussion section, we give a putative interpretation of this difference.

Although free association paradigms have been used in various previous studies to operationalize the psychodynamic construct of repression, they have several shortcomings in this respect. First, as they do not include a measure of consciousness, these paradigms do not allow one to test if forgetting indeed occurs unconsciously, as would be demanded from the viewpoint of psychodynamic theory. Second, it is unclear if the freely generated words are actually related to internal conflicts. Thus, the free association paradigms in their current form are not sufficiently well controlled to make sure what they are actually investigating is a process that is closely related to the psychodynamic construct of repression. We were nevertheless interested to see if application of these paradigms in a neuroimaging context provides results that are at least consistent with repression theory. We speculate that one day, revised versions of these paradigms – e.g., testing simultaneously several measures of both conscious and unconscious memory/forgetting processes, and using individualized stimuli in clinical populations [35] – may induce processes that are more closely related to the psychodynamic construct of repression. Our current efforts are definitely only a first step in this direction.

In detail, we tested whether an adaptation of free association paradigms in two functional MRI (fMRI) experiments indeed reveals results which are consistent with the hypotheses of activation of internal conflicts and suppression of conscious memory systems. Therefore, we expect to find increased blood oxygenation level dependent (BOLD) responses in the ACC (related to conflicts) and decreased activity in regions of the medial temporal lobe (related to reduced conscious memory) during putatively conflict-related conditions. In addition, we tested the alternative explanation that subsequent forgetting can only be explained by the cognitive difficulty of word generation. We thus calculated whether response entropy (related to the number of different words that were generated to each cue by the group of participants) fully explains the effect of arousal on subsequent memory. Another alternative hypothesis, that subsequent memory can be explained by semantic similarity between stimulus word and generated association, was also investigated using a measure of semantic similarity based on distributional similarity of words (see Methods).

## Methods

### Ethics Statement

The study was approved by the local medical ethics committee (“Ethikkommission an der Medizinischen Fakultaet der Rheinischen Friedrich-Wilhelms-Universitaet Bonn”), was according to the latest version of the Declaration of Helsinki, and all subjects provided written informed consent.

### Participants

Participants were recruited through notifications on the homepage of University Bonn Students’ Service. They were paid 10€ per hour (total time for the experiment 3.5–4 hours). They were right-handed, native German speakers with normal or corrected-to-normal vision and without current or past neurological or psychiatric diseases.

*For the first experiment*, we scanned 27 subjects, 5 of which were afterwards excluded from fMRI analysis because of excessive motion artifacts (more than one voxel diameter, due to the overt speech in the scanner, see below; 4 subjects), or technical problems with the presentation program (1 subject). 6 subjects were excluded from behavioral and SCR data analysis due to deficient skin conductance recordings (5 subjects) or technical problems with the presentation program (1 subject). Of the 26 subjects included in fMRI and/or behavioral/SCR analysis, 13 were females. The mean age of these 26 participants was 24.2±3.1 years (mean ± standard deviation).

*For the second experiment*, 23 subjects were scanned, 5 of which were excluded from fMRI analysis because of high motion artifacts (more than one voxel diameter; 3 subjects), or early interruption of the experiment (2 subjects). Two subjects were excluded from behavioral and SCR analysis because of early interruption of the experiment. Of the 21 participants included in fMRI and/or behavioral/SCR analysis (11 female), mean age was 25.7±3.2 years.

### Experimental Paradigms

#### First paradigm

The first experiment consisted of 4 parts: association phase, break/distraction, memory recall, and rating. On arrival, subjects were given safety instructions concerning MRI and were given instructions for the association phase of the experiment in written form (these instructions are provided in Table S1 and Table S2). While already inside the scanner, participants had the opportunity to become acquainted with the association paradigm by completing a short practice version, consisting of 6 stimulus words. Two electrodes for SCR recording were attached to the subjects’ left palm (thenar and hypothenar), and an MRI-compatible microphone was positioned in front of the lips for audio recording (Fibersound® Microphone Model FOM1-MR and Fibersound® Control Model FOM1-DRx Battery/wall powered; Micro Optics Technologies Fibersound™ Audio, Middleton, USA). The paradigm was presented via MRI-compatible video goggles (Nordic Neuro Lab, Bergen, Norway). We scanned the subjects during retrieval as well as during the free associations phase to maximize similarity between the word generation and the retrieval condition, which should facilitate retrieval according to transfer appropriate processing theory [36].

Association phase: After a fixation cross varying in length from 1.5 to 3 s, the stimulus word was presented for 1 second, followed by a question mark for 10 s, indicating the request to generate a single word association. Starting with stimulus presentation, the subjects’ verbal response was recorded for 11 seconds per trial (up to the end of the question mark). The stimulus list consisted of 150 German nouns with a moderate frequency of occurrence (112/10.000.000 as indicated by CELEX), and was presented in random order.

Break/distraction: The first part of the experiment was followed by a 1-hour break, during which subjects completed the German version of the defense style questionnaire 40 (DSQ-40) [37]. The DSQ-40 is a questionnaire where subjects can assess on a 9-point Likert scale the degree to which various statements apply to them, which can then be grouped and attributed to maladaptive, adaptive or neurotic defense mechanisms.

Memory recall: After the break, participants were given written instructions for the unexpected recall of their previous associations. Again, they laid down in the MRI and were connected to the SCR-amplifier. All 150 words from the list were again presented in random order for 1 s, followed by a period of 10 s duration to remember and enunciate the association that was given in the first part of the study. Again, the response was recorded digitally. Before the presentation of each new stimulus, there was a random inter-stimulus interval of 1.5–3 s duration, during which a fixation cross was presented. Subjects were only allowed to give one answer per stimulus, and were encouraged to make a guess if they were not sure about their answer. To enhance their motivation and efforts, each correct answer was rewarded with 0.10€, and each incorrect answer was “punished” by losing 0.05€. Since audio recordings of association and recall had to be compared individually (by listening to them) in order to check the correctness of the memory recall, the subjects received no direct feedback whether their recall was correct or not.

Rating: In the last part of the experiment, subjects were asked to rate all 150 stimulus words on two 9-step Likert scales, according to their valence (“Is the presented word POSITIVE or NEGATIVE to you?”, with a scale ranging from −4, very negative, to 0, neutral, and +4, very positive) and the level of arousal they elicited (“How strong is your feeling accompanying the word?”, with a scale ranging from 1, slight, to 5, moderate, to 9, very strong). Ratings were conducted outside the MRI scanner on a laptop computer.

#### Second paradigm

In brief, the second experiment involved three important modifications: First, associations were not cued by words, but by entire sentences. These sentences were either neutral, negative but unrelated to conflicts, or related to typical conflicts regarding “desire for care vs. autarchy” or self-value (for a full list of the sentences we used, see Table 1). Second, as the number of trials was lower in this paradigm (24 sentences instead of 150 words in the first experiment), subjects were asked to name the first *three* words which came into their minds after presentation of each cue to avoid ceiling effects during subsequent memory recall. Finally, trials lasted for 60 seconds following offset of each stimulus and included free association after generation of the first three words: After stimulus presentation, subjects had to name the first three words that came to their mind and use the remainder of the 60 seconds for free association, which was also recorded via microphone.

**Table 1:** List of sentences used in the second paradigm.

**neutral sentences**
Occasionally I like to watch movies on the television.
I try to follow the news on a regular basis.
Sometimes my mood is influenced by the weather.
There are topics I am more interested in than politics or economy.
Mostly I do respect the traffic regulations.
I find it important to find the time for my hobbies once in a while.
**negative sentences**
I am getting annoyed when I am stuck in a traffic jam and I have an important appointment.
Sometimes I am frightened when I walk alone in the dark.
When an overtaking car on the other side of the street approaches me, my heart sinks into my boots.
Sometimes I become sad, when I think about dead soldiers in the war.
Seeing a helpless animal suffer often makes me sad.
When somebody is pushing in the line, it can really upset me.
**conflict sentences: desire for care vs. autarchy (passive)**
All my life I got a raw deal.
I wish that finally someone is taking care of me.
I have the feeling that I always get too little.
I actually only feel good when someone is taking care of me.
**conflict sentences: desire for care vs. autarchy (active)**
I give so much, without getting really rewarded.
I cannot say “No” if someone else is asking me for help.
I do not need nothing or anybody to be happy.
I hate it to be a burden for other people.
**conflict sentences: self-value**
Usually I have a very low self esteem.
I am often embarrassed about myself.
Sometimes I am disgusted by myself.
I often estimate myself as little competent.

All participants of the second study were invited a few days before the experiment in order to practice the technique of free association and to be screened for current existence of psychiatric symptoms. For this screening, two questionnaires were used: SCL-90 (symptom check list) and BDI (Beck’s Depression Inventory). Those who scored high on either of the questionnaires (cut-offs: BDI>11, SCL-90 GSI>0.57) were excluded from the experiment.

The second paradigm consisted of 3 parts: association phase (in this experiment also including ratings of valence, arousal and agreement with each sentence), break/distraction, and memory recall. Before the beginning of the association phase, subjects were given MRI safety instructions and received written instructions for the first part of the experiment.

Association phase: Subjects were placed in the MRI scanner, with video goggles to present stimuli, a microphone to record verbal response, and two electrodes connected to the right palm for SCR measurements. A hand-held four-button device was used for rating. A stimulus (one of 24 sentences, presented in random order) was shown for 5 seconds, followed by 60 s time period (indicated by a question mark) for free association. During this total period of 65 seconds, the verbal responses of the subjects were digitally recorded. The participants were asked to say the first three words that came to their mind after stimulus presentation, and use the remaining time for (spoken) free association. Afterwards, subjects rated their agreement with the sentence (on a scale ranging from 1: very strong disagreement to 9: very strong agreement), and their own emotional state after association in terms of valence (−4: very negative to +4: very positive feeling) and arousal (1: very calm to 9: very aroused). Rating was followed by a 30-second break. After an inter-stimulus-interval (fixation cross) of 1.5 to 3 seconds, the next stimulus was presented.

Of the 24 stimulus sentences, 6 were „neutral“ and 6 were „generally negative“, while the remaining 12 were „conflict related“, meaning that they were designed to resemble typical expressions of common intrapsychical conflicts. Those conflict types were selected on the basis of specific conflicts as defined in OPD (operationalized psychodynamic diagnostics), a German-language diagnostic tool for intrapsychical conflicts. Two types of conflict were used for the generation of conflict-related sentences: autonomy/dependency, and self-esteem–conflict.

Break/distraction: Following the association-phase, there was a 1-hour break, during which participants filled out the DSQ-40 questionnaire.

Memory recall: After the break, subjects had to perform an unexpected memory recall task. Again, they were placed in the MRI scanner with video goggles, microphone and SCR-electrodes. All 24 sentences were presented again, after each of which subjects had 30 seconds to remember and name the 3 words that had come to their mind to that sentence in the beginning of the association phase in the first part of the experiment (not the content of the following free association phase). Again, answers were recorded via microphone. Only the first 3 answers were evaluated, and participants were encouraged to guess if they were unsure. As in the first paradigm, subjects were rewarded with 0.10€ for each correct answer afterwards, and for each incorrect or missing answer 0.05€ were subtracted from their total gain.

### General Considerations on the Analysis of Repression Effects

Both for the analysis of fMRI and SCR data, we expected repression effects to occur relatively early after cue presentation (i.e., within around one second). Although this assumption has to remain somewhat speculative, it is based on the idea that cues trigger internal conflicts rapidly, and before a participant is able to generate a word by free association, which is based on implicit conceptual memory (see Discussion). This is reflected both in the timing of the regressors used for fMRI analysis (delta pulses triggered to the onset of cue word presentation in the first experiment, cue sentence presentation in the second experiment) and in the timing of the intervals used for SCR analysis: SCR pulses typically peak around 4–5 seconds after the physiological event that caused them. Therefore, to investigate processes occurring early after cue presentation, an analysis of SCR amplitudes in this interval should be appropriate.

In our data, the intervals used for analysis of SCR data were derived from the activation maxima of SCR curves averaged across conditions (to avoid any bias). In experiment 1, this grand average curve peaked at 5.0±3.7 seconds (mean±standard deviation of all trials) after stimulus presentation. We selected an interval of mean±0.5 standard deviations (5.0 s±1.85 s = 3.15 s–6.85 s after cue presentation) to allow for some inter-trial variability.

In experiment 2, condition-averaged SCR curves peaked at 8.8±8.6 seconds after stimulus presentation. This more delayed response may be due to a more persistent increase of physiological arousal following sentence presentation. Again, we chose an interval of mean±0.5 standard deviations (8.8 s±4.3 s = 4.5 s–13.1 s after cue presentation) for analysis.

### MRI Data Acquisition and Analysis

Thirty-four axial slices were collected at 1.5T (Avanto, Siemens, Erlangen, Germany). We collected T2*-weighted, gradient echo EPI scans (slice thickness: 3.0 mm; voxel size: 3×3×3 mm; matrix size: 64×64; field of view: 210×210 mm; repetition time: 2700 ms; echo time: 40 ms). Thereafter, we acquired a 3D-sagittal T1-weighted MPRAGE sequence for each subject for anatomical localization (number of slices: 160; slice thickness: 1 mm; inter-slice gap: 0.5 mm; voxel size: 1×1×1 mm; matrix size 256×256; field of view: 256 mm; echo time: 3.09 ms; repetition time: 1660 ms).

As described in the Introduction, we attempted to investigate whether free association may overcome repression, leading to generation of words which are possibly related to internal conflicts, which may induce autonomic arousal and new repression (impairing subsequent conscious access). Therefore, we only analyzed activity during the free association phase of the experiment, but not during the recall phase.

MRIs were pre-processed in SPM5 (http://www.fil.ion.ucl.ac.uk/spm/) using standard pre-processing steps including realignment, unwarping, normalization, and smoothing with a 6-mm Gaussian kernel. Pre-processed data were fitted by the convolution of multiple regressors with a canonical hemodynamic response function to obtain parameter estimates for each condition covariate.

The following set of regressors was used: In the *first paradigm*, we used two event-related (delta-pulse) regressors for subsequently forgotten and subsequently remembered words (triggered to the onset times of cue word presentation), two associated regressors with temporal derivatives of the canonical hemodynamic response function (to account for slight temporal shifts in the onset of the BOLD responses), a linear slope to model slow scanner drifts, and one regressor to model the mean activation. To rule out the potential effect of cognitive effort, response entropy (see below) was added as a parametric modulation to the regressors for subsequently forgotten and subsequently remembered words. However, inclusion of response entropy had little effect on activation patterns (in the following, all fMRI results reported relate to the model which includes response entropy). In the *second paradigm*, we used separate event-related (delta-pulse) regressors (triggered to the onset times of cue word presentation) to model activity during the neutral, negative and conflict conditions, one additional regressor to model the rating periods after each free association period, one regressor for the inter-stimulus break, and two regressors for linear scanner drift and mean activation. Analogous to the first experiment, response entropy was added as a parametric modulation of the regressors for neutral, negative, and conflict sentences (again, with no significant differences compared to a model without response entropy). Parametric modeling of emotion rating did not have an impact on the pattern of BOLD activation and was thus not considered in further analyses. Finally, we calculated an additional model in which the number of subsequently remembered words was used as parametric modulator.

In both paradigms, figures with fMRI results are displayed using neurological convention (left hemisphere on the left side of the figure). To identify significant activations, we used an uncorrected voxel threshold of P<0.001 and an additional cluster threshold of p<0.05, corrected for multiple comparisons using the false discovery rate (FDR) procedure of SPM5.

### Analysis of Reaction Times

As described above, word associations were generated by freely speaking inside the scanner and recorded using an MR-compatible microphone. Audio recording was started simultaneously with stimulus presentation and continued until the end of the association period (amounting to a recording length of 11 seconds per trial for the first paradigm, and 65 seconds for the second paradigm). The responses were then digitized and the acoustic waveforms were transformed into visual traces using “Audacity” free audio editor software (version 1.3.12; http://audacity.sourceforge.net/). Within these visual traces, the onset of the generated word was detected manually. For the second paradigm, only the onset of the first generated word was identified and used as reaction time.

### SCR Acquisition and Analysis

We collected the SCR data with a sampling rate of 1000 Hz with BrainVision Recorder Software. Data were corrected for MRI-artifacts using BrainVision Analyser 2.0. We down-sampled data to 200 Hz and low-pass filtered them at 5 Hz. The corrected data were analyzed using LEDALAB [38] to extract phasic electrodermal activity in an integral of 3.15 s–5.85 s (first paradigm) or 4.5 s–13.1 s (second paradigm) after stimulus presentation. Different intervals were chosen because of the prolonged word generation period in the second paradigm, the longer presentation time of the sentences (5 s versus 1 s for the individual cue words in the first paradigm), and different average peak times for SCR in the different paradigms. However, we chose the same criterion (mean±0.5 standard deviations) for both paradigms. For descriptive purposes, we plotted the SCR-curves with MATLAB using as a baseline the mean conductance in the interval between −200 and 0 ms prior to stimulus presentation.

### Statistical Analysis

We conducted one-tailed tests if we had directed a priori expectations and two-tailed tests otherwise. In detail, we had the following directed hypotheses based on the results from previous studies [25]–[27]:

### First Experiment

Longer RTs (and higher SCRs) for subsequently forgotten as compared to remembered words
Positive correlation between RTs and SCRs
Higher response entropies for words that were subsequently forgotten as compared to remembered words, and for words which were associated with longer RTs (and higher SCRs).

### Second Experiment

Longer RTs (and higher SCRs) for trials with less remembered words (i.e., negative correlations between RTs (and SCRs) and the number of remembered words per trial)
Positive correlation between RTs and SCRs
Better memory for associated words in the negative (or neutral) condition as compared to the conflict condition
Longer RTs (and higher SCRs) in the conflict as compared to the negative (or neutral) condition
Higher response entropies for trials with a higher number of subsequently forgotten words, and for trials which were associated with longer RTs (and higher SCRs).

### Analysis of Response Entropy

We analyzed response entropy similar to Levinger and Clark [25] with a slight modification: Instead of quantifying response entropy by the number of different responses, we calculated the Shannon entropy [39] for each cue word by the variance of the distribution of responses:
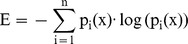
with *n* being the number of subjects (and thus of possible different responses), *p_i_(x)* denoting the probability of occurrence of each possible response. The reason for this was that the measure of response variability used by Levinger and Clark would not distinguish, e.g., between two different responses each given by half of all participants and two different responses, where one is given only by a single participant and one by all other participants, whereas our measure of entropy is sensitive to this difference.

### Analysis of Semantic Similarity

To investigate whether subsequent memory for associations could be explained by semantic similarity between stimulus word and generated association, we conducted an analysis of semantic similarity similar to latent semantic analysis [40], [41] using Linguatools’ DISCO-software (www.linguatools.de/disco [42]). DISCO is based on a German language database that consists of encyclopedia entries, newspaper and magazine articles, parliamentary debates, and works of literature, and provides two different measures of semantic relationship: “semantic similarity” and “semantic relatedness”. Both are calculated based on distributional similarity of words within the database. To compare two words, a set is calculated for each word which consists of words that co-occur most commonly with this word. The two sets are then compared to determine semantic similarity (between 0 and 1).

## Results

### First Experiment

**Figure 1:**
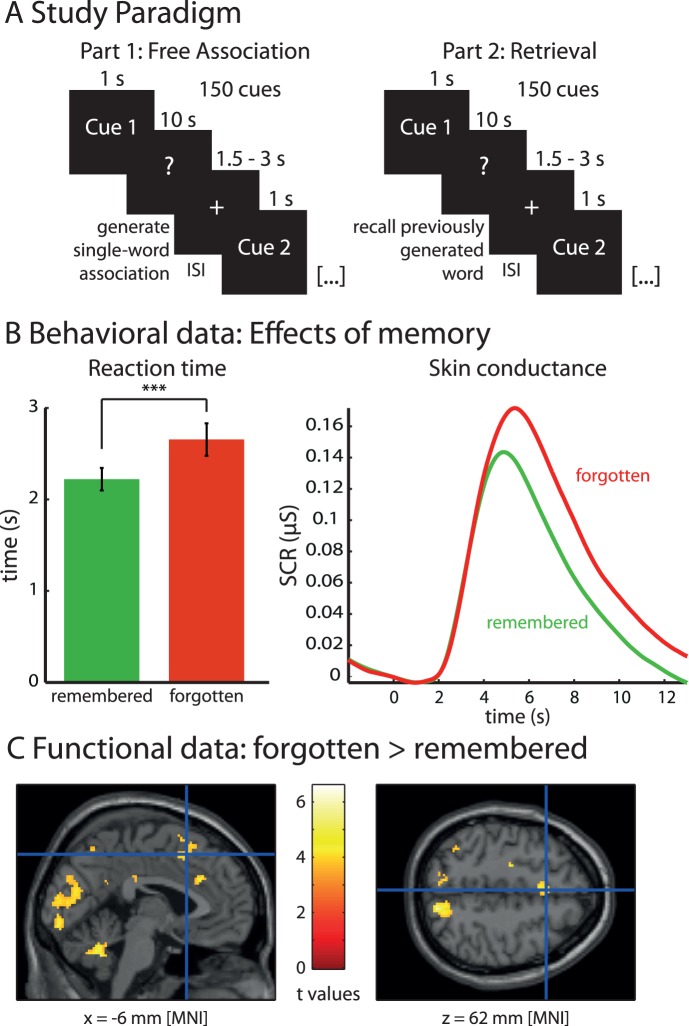
Neural correlates of forgetting arousing information. (A) Experimental design of the first experiment. Part 1 depicts one trial of the association task. A cue word is presented for 1s, and then participants are asked to freely generate an association. During retrieval (part 2), the same list of cue words is presented, and subjects are asked to recall the previously associated words. (B) Subsequently forgotten words are generated with a higher reaction time and a larger skin conductance response. (C) Functional data of the contrast forgotten versus remembered word associations indicate increased activation of the anterior cingulate cortex/pre-supplementary motor area during the free association phase for subsequently forgotten words (cluster threshold of p_FDR_<0.05). All bar plots indicate mean values with S.E.M.

In our first experiment, we measured SCR and BOLD responses while participants generated free associations to a list of words, and subsequently tested memory for these associations (Fig. 1A). Of the generated associations, 24.3%±10.8% (mean ± standard deviation) were not remembered subsequently. We first analyzed whether free association of subsequently forgotten words occurs with high arousal and activates brain regions related to conflict processing. Indeed, generation of subsequently forgotten words was associated with increased SCRs (t_20_ = 2.50; p = 0.011) and longer RTs (t_20_ = 6.76; p<10^−6^), compared to subsequently remembered words (Fig. 1B). SCRs and RTs were highly correlated (mean of Spearman’s Rs: 0.31; t_20_ = 6.11; p<10^−5^ [t-test of Fisher-z-transformed Spearman’s R-values tested against 0]). Functional MRI data showed that subsequent forgetting is associated with activation of the ACC/pre-supplementary motor area (pre-SMA) (MNI coordinates: −6/12/62; Fig. 1C; see Table 2 for an overview of all significant activations at a threshold of p_FDR_<0.05). No region showed significant activation at this threshold in the reverse contrast.

**Table 2:** Overview of significant activations (p_FDR_ <0.05).

MNI							
x	y	z	cluster size	t value			Brodmann areas
**First experiment**							
**Forgotten vs. remembered words**							
16	−66	44	778	6.58	precuneus		BA 7, BA 19, BA 18, BA 31
−6	−58	−26	140	6.20	cerebellum		–
−16	−80	12	1031	5.85	cuneus,		BA 18, BA 17, BA 19, BA 31,
					calcarine fissure		BA 23, BA 30
−6	12	62	161	4.67	pre-SMA/ACC		BA 8, BA 6, BA 32
**Remembered vs. forgotten words**							
No suprathreshold activation							
**Second experiment**							
**Conflict-related sentences vs. negative sentences**							
−48	−40	46	363	6.66	inferior parietal lobule		BA 40, BA 2
48	−50	−2	143	6.64	middle temporal gyrus		BA 22
44	−50	46	428	6.63	inferior parietal lobule		BA 40, BA 7
44	−12	16	93	6.61	insula (rolandic operculum)		BA 13, BA 43
−6	4	48	307	6.13	pre-SMA/ACC		BA 24, BA 6, BA 32
16	34	46	129	5.85	medial frontal gyrus, ACC		BA 9, BA 32
30	10	38	98	5.28	middle frontal gyrus		BA 8
−48	−20	40	99	5.20	postcentral gyrus		BA 3, BA 4, BA 2
−8	−72	42	112	4.81	precuneus		BA 7
**Negative vs. conflict-related sentences**							
10	−58	14	120	6.30	posterior cingulate, precuneus		BA 23, BA 30
**Negative vs. neutral sentences**							
No suprathreshold activation							
**Neutral vs. negative sentences**							
−52	−44	32	181	6.30	supramarginal gyrus		BA 40
−34	−16	40	605	6.06	precentral gyrus,		BA 6, BA 4, BA 3, BA 9, BA 2
36	−12	34	214	5.76	postcentral gyrusprecentral gyrus		BA 6
38	−48	32	255	5.63	inferior parietal lobule		BA 40, BA 2
32	−64	48	132	4.77	superior parietal lobule		BA 7, BA 39, BA 19, BA 40
**Conflict-related vs. neutral sentences**							
No suprathreshold activation							
**Neutral vs. conflict-related sentences**							
−18	−36	−14	347	6.50	hippocampus,		BA 37, BA 36, BA 27, BA 35,
					parahippocampal cortex		BA 30, BA 28
−18	−78	−10	163	5.89	lingual gyrus		BA 18, Cerebellum
−16	−54	10	324	5.72	posterior cingulate, calcarine		BA 30, BA 23, BA 29, BA 18,
					fissure, precuneus		BA 19, BA 31
14	−58	16	163	5.01	posterior cingulate, calcarine		BA 29, BA 30, BA 31, BA 19
					fissure, precuneus		

Only the most strongly activated voxel in each significant cluster is indicated.

Further analyses were conducted to test if this effect can be explained by cognitive processes such as the difficulty of generating an associated word or the perceived valence or arousal of the presented words. We found that forgetting could not be solely explained by the difficulty of word generation on a cognitive level as assessed by response entropy: Response entropies were significantly correlated with memory (Spearman’s r = −0.30; p<0.001), RTs (r = 0.49; p<0.0001) and SCRs (r = 0.36; p<10^−5^), indicating that more variable associations were associated with longer RTs and higher SCRs and predicted subsequent forgetting. Importantly, a partial correlation analysis showed that even when response entropy was partialed out, RTs (r = −0.34; p<0.0001) and SCRs (r = −0.18; p = 0.016) were significantly correlated with memory. Next, we investigated whether semantic similarity and semantic relatedness were correlated with subsequent memory. We calculated semantic similarity and semantic relatedness between the cue words and the generated associations (for 147 out of 150 cue words that were found in the database). Spearman’s rank correlation between mean subsequent memory for each word and mean similarity (r = 0.054, p = 0.51) or relatedness (r = 0.004, p = 0.96) showed no significant correlations between similarity or relatedness and memory. Additional analyses showed that memory was not influenced by word frequency (r = 0.08; p = 0.339; calculated across 148 out of 150 words for which normative data could be obtained from CELEX) or word length (r = −0.11; p = 0.201).

Furthermore, conscious ratings of emotional valence and arousal of each item were not predictive for memory: There were no differences between cue words associated with subsequently forgotten as compared to subsequently remembered associations, neither in terms of valence (t_20_ = 1.322; p = 0.201), nor in terms of arousal (t_20_ = 1.076; p = 0.295).

### Second Experiment: Behavioral and SCR Data

**Figure 2:**
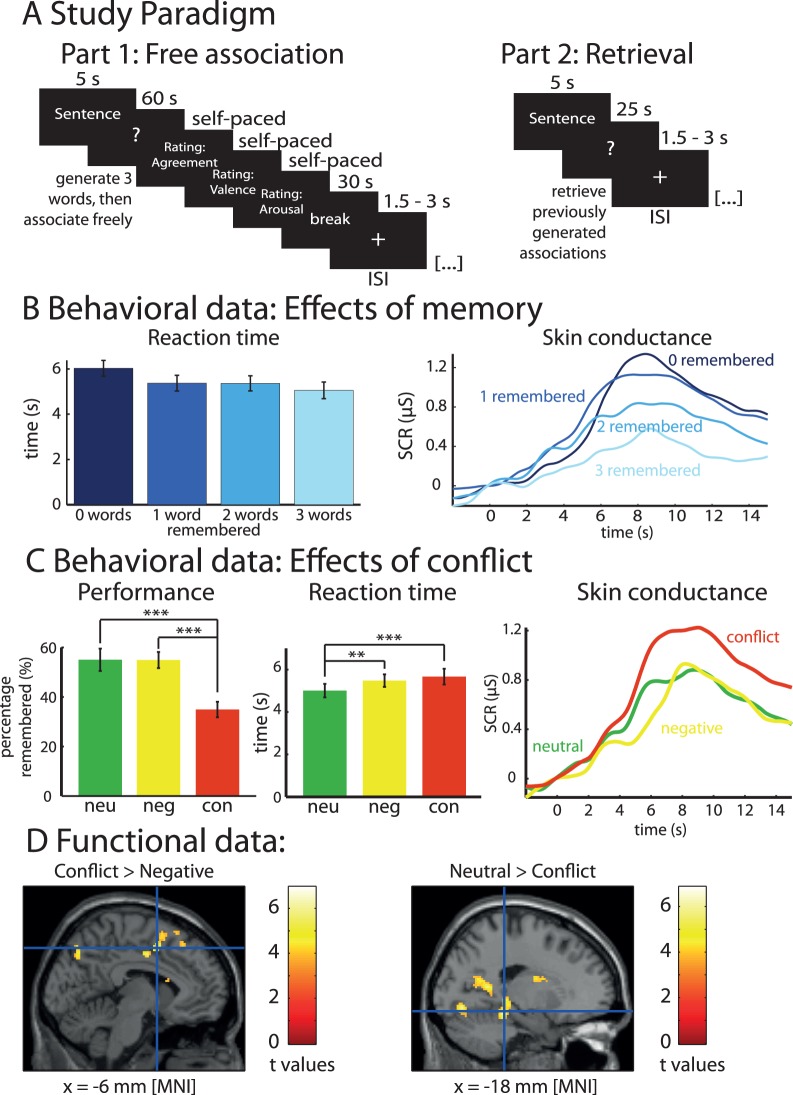
Associations to conflict-related sentences. (A) Experimental design of the second experiment. In this study, participants name three words and then associate freely for a longer period of time (60 s) following presentation of neutral, negative but not conflict-related and conflict-related sentences (part 1). During retrieval, they are asked to recall the three words generated during the free association. (B) Higher reaction times and skin conductance response during free association predict subsequent forgetting. (C) Free associations to conflict-related words are forgotten more often than associations to either negative or neutral sentences (left), are given with longer reaction times (middle) and accompanied by higher SCRs (right). (D) Activation of the anterior cingulate cortex/pre-supplementary motor area and deactivation of the hippocampus and parahippocampal cortex during associations to conflict-related sentences. All bar plots indicate mean values with S.E.M.

Next, we conducted a second experiment in which possible conflicts did not emerge spontaneously during presentation of individual words, but were directly induced by conflict-related sentences (i.e., “conflict” was an independent variable; Fig. 2A). Consistent with our first experiment, we found that free association of subsequently forgotten words was accompanied by higher SCRs and RTs as compared to free association of subsequently remembered words (Fig. 2B): There were negative (intra-individual) correlations of the number of subsequently remembered words with SCR (mean of Spearman’s Rs: −0.09; t_20_ = 2.36; p = 0.014 [t-test of Fisher-z-transformed Spearman’s R-values tested against 0]) and RT (mean of Spearman’s Rs: −0.14; t_20_ = 3.31; p = 0.002). Again, RTs and SCRs were significantly correlated (mean of Spearman’s Rs: 0.15; t_20_ = 4.05; p<0.001).

As for the first experiment, we analyzed whether effects could be explained by response entropies. We found that response entropies were significantly correlated with memory (Spearman’s r = −0.40; p = 0.026) and RTs (r = 0.43; p = 0.019), and in trend with SCRs (r = 0.29; p = 0.087). Again, partial correlation analysis showed that there was still a significant effect of RTs on memory (r = −0.37; p = 0.040) and SCRs on memory (r = −0.41; p = 0.027) after partialing out the effect of entropy.

In a second analysis step, we looked at differences related to sentence type (neutral, negative, or conflict-related; Fig. 2C). Associations to conflict-related sentences were forgotten more often (65±3.11% forgotten words) as compared to associations to neutral (45±4.53%; t_20_ = 6.90; p<10^−6^) or negative sentences (45±3.25%; t_20_ = 6.45; p<10^−5^). Conflict-related sentences showed significantly longer RTs compared to neutral sentences (t_20_ = 5.45; p<10^−4^), while the difference in RTs between conflict-related and negative sentences was not significant (t_20_ = 1.28; p = 0.11). Furthermore, SCRs to conflict-related sentences were higher as compared to negative (t_20_ = 2.40; p = 0.013) or neutral sentences (t_20_ = 2.25; p = 0.018) (Fig. 2D).

Next, we investigated whether there was an interaction between memory and sentence type for RT (and SCR), because such an interaction might be an argument to indeed interpret longer RT (and SCR) as a marker of repression. Indeed, we observed such an interaction for reaction times, indicating that longer RTs were only related to forgetting in the conflict condition, but not in any other condition. In detail, we performed the same analysis (intra-individual Spearman rank correlations between memory and RT) as before, but now separately for the different sentence types. Spearman’s Rs of the individual participants were then Fisher-z-transformed and entered into a one-way ANOVA with “sentence type” as repeated measure. This ANOVA revealed a significant effect of “sentence type” (F_2,40_ = 3.771, p = 0.032), indicating different correlations between RT and memory for the three conditions. Next, we tested whether correlations were significantly different from zero in each of the three conditions by calculating t-tests of Fisher-z-transformed Spearman’s Rs against zero. We found that correlations were significantly different from zero only for conflict sentences (mean r = −0.228, p = 0.0027), but not for neutral sentences (mean r = −0.004, p = 0.88) or for negative sentences (mean r = 0.123, p = 0.20).

The same analysis also showed a significant difference in correlation values between SCR and memory as a function of sentence type (F_2,40_ = 3.55, p = 0.038), indicating different correlations between SCR and memory for the three conditions. Next, we tested whether correlations were significantly different from zero in each of the three conditions by calculating t-tests of Fisher-z-transformed Spearman’s Rs against zero. We found that correlations were significantly different from zero only for conflict sentences (mean r = −0.12, p = 0.020), but not for negative sentences (mean r = 0.12, p = 0.845) and only in trend for neutral sentences (mean r = −0.13, p = 0.063).

Then, we tested whether differences between conditions could be explained by the conscious ratings of arousal or valence given after each association trial. Valence was highest for neutral sentences and lowest for negative sentences, with conflict sentences in-between them (neutral: 1.08; conflict: 0.37; negative: −0.19). Differences were significant between neutral and conflict sentences (t_20_ = 4.00; p<0.001), between neutral and negative sentences (t_20_ = 7.18; p<10^−6^), as well as between negative and conflict sentences (t_20_ = 2.21; p = 0.039). Concerning arousal, there was no significant difference between the categories (neutral: 5.65; conflict: 5.43; negative: 5.82; all t_20_<1.63; all p>0.11). Self-rated valence did not predict subsequent memory (mean of Spearman’s Rs: 0.01; t_20_ = 0.252; p = 0.8 [t-test of Fisher-z-transformed individual Spearman’s R-values tested against 0]). Similarly, forgetting could not be explained by higher arousal during free association, as higher levels of self-rated arousal even predicted *better* subsequent memory (mean of Spearman’s Rs: 0.14; t_20_ = 3.329; p<0.01). In addition to valence and arousal, we asked participants to rate the degree of consent to each sentence. Consent was lowest to conflict sentences (mean: 3.39) as compared to either negative (mean: 5.37; t_20_ = 5.74; p<10^−4^) or neutral sentences (mean: 6.05; t_20_ = 9.08; p<10^−7^). Not surprisingly, higher consent with the content of a sentence predicted subsequent memory (mean of Spearman’s Rs: 0.20; t_20_ = 4.236; p<0.001). To investigate whether the correlations of RT and SCR with memory can be explained by consent, we conducted a partial correlation analysis in which we correlated (across all sentences, and separately for each participant) RT and SCR with memory and added “consent” as additional variable. These analysis showed that memory was still significantly correlated with RT (mean R: −0.13, p = 0.0016) and SCR (mean R: −0.10, p = 0.008) even after partialing out consent.

Finally, increases of SCR values during conflict-related as compared to neutral sentences were associated with relatively more maladaptive defense mechanisms (r = 0.52; p = 0.019). Although our study participants as a group showed clearly little maladaptive defense mechanisms, the correlation of relative differences in defense and SCR during conflict-related sentences could contribute to an external validation of assumed repression in our experiment.

### Second Experiment: fMRI Data

In the fMRI data, we calculated a different contrast as compared to the first study because trials could not be unequivocally distinguished according to subsequent memory for the associated words (there could be either 0, 1, 2, or 3 remembered associated words). An alternative model in which all trials were attributed to a single regressor and parametrically modulated by the number of subsequently remembered words showed no significant clusters of activation in either direction. Therefore, we contrasted activity during conflict-related sentences as compared to negative or neutral sentences. We found that conflict-related sentences were associated with increased activity of the ACC/pre-SMA as compared to negative sentences (MNI coordinates: -6/4/48 and 16/34/46; Fig. 2E), and with deactivation of left hippocampus and parahippocampal cortex as compared to neutral sentences (−18/−36/−14).

## Discussion

In brief, we found that the spontaneous occurrence (experiment 1) or induction (experiment 2) of autonomic arousal during free association led to subsequent memory failure and was associated with increased activation of the anterior cingulate cortex. In both experiments, activations were within the anterior dorsal ACC (adACC), which shows rich connectivity with limbic regions (i.e., amygdala, periaqueductal gray and hypothalamus) and plays a major role in emotional processing [15]–[19], [22], although ACC activations in the second experiment were slightly more posterior and ventral as compared to the first experiment. These results are consistent with the hypothesis that free association of words re-activates internal conflicts, which generates autonomic arousal and impairs subsequent conscious memory access.

Interestingly, we observed that the same contrasts which yielded activation in the adACC were also associated with increased BOLD responses in the visual cortex. This result is consistent with previous findings that salient visual stimuli enhance activity in the visual cortex via a top-down control to facilitate processing of these stimuli [43].

### Interpretation Issues

Reaction times are obviously a very indirect and unspecific measure of any psychological processes. Therefore, any interpretation of emotional effects underlying long reaction times has to remain speculative. In the following, we would like to describe this speculative background, knowing that only a series of careful experiments which aims at operationalizing clinical constructs will allow to link these constructs to reaction times (as well as to other physiological measures and to neural activation patterns).

In essence, we argue that it is the cue word and not the generated word which is responsible for effects on reaction times. We have followed the interpretation of previous researchers who used the free association paradigm that long reaction times are a measure of repression [23], [25]–[27]. According to this line of argumentation, repression occurs because the content of a presented cue word (or, in the second experiment, of a cue sentence) is associated with an internal conflict. In many cases, it is likely that this association is due to an idiosyncratic association, and is most likely not consciously perceived.

We do not argue that the content of the freely generated words is related to an internal conflict: When a cue word triggers (through association) memory of a significant conflict, participants may or may not respond with a word which is related to this conflict. Already Jung noted that the generated words are not direct expressions of psychological associations, but only “symptoms” of them [44]. In other words, the generated associations do not necessarily describe relevant conflicts; however, the circumstances under which these associations are generated may allow one to draw (speculative) inferences on the underlying psychological processes. Our data only indicate that after long reaction times (and high SCRs), word generation occurs during cognitive/affective conditions which are unfavorable for later conscious recall.

The relationship between reaction times and repression is supported by one finding of the second experiment: We observed that longer RTs were related to subsequent forgetting specifically for conflict sentences, but not for negative and neutral sentences. The same holds true for SCRs. Although we are hesitant to over-interpret this finding, it appears to indicate that longer reaction times and increased SCRs during free associations do not generally lead to later forgetting, but specifically so for conflict-related cues.

Not only our behavioral measures, but also the fMRI data only allow one to draw indirect and relatively speculative inferences. Activation of the adACC in fMRI experiments does not necessarily imply that internal conflicts were involved. In general, although reverse inference (i.e., inference from a given brain activation pattern to an underlying psychological process) is the common logic of most fMRI studies, this reasoning is flawed by the usually relatively low specificity of regional BOLD responses (e.g., [45]–[47]). Therefore, our findings should only be viewed as consistent with, but not as conclusive evidence for, a role of conflict in the regulation of memory in our paradigm.

### Relationship to Previous Studies on the Role of Emotion, Arousal and Cortisol on Memory

It is well established that emotionally negative stimuli such as International Affective Picture System (IAPS) pictures [24] or faces with negative expressions are better remembered than neutral items [29]–[32]. Autonomic arousal is associated with increased levels of noradrenaline and cortisol, which may facilitate learning processes in regions supporting declarative memory processes such as the hippocampus [48]. However, further enhancements of autonomic responses can actually severely deteriorate declarative memory processes, leading to an inverted-U-shape relationship between arousal and declarative memory. Hippocampal cortisol receptors with different affinities appear to constitute the physiological basis for this effect: Moderate increases of cortisol concentrations primarily activate highly affine mineralocorticoid receptors in the hippocampus and subsequently facilitate synaptic long-term plasticity, the putative cellular correlate of learning and memory formation [48], [49]. If the concentration of cortisol increases further, however, also glucocorticoid receptors with low affinity are activated, which impairs hippocampal functioning [48]. This is the case, for example, during exposure to traumatic experiences, which – in addition to hypermnestic symptoms such as spontaneous intrusions and flashbacks – may lead to partial or complete amnesia for traumatic events, in particular after peritraumatic dissociation [50]. Similar effects may occur during events which lead to repression, because they are related to an unbearable conflict (e.g., [12]), or during cues which induce associations of repressed contents. The results of our study are consistent with the idea that some of the words presented during the word-list paradigm (experiment 1) or (with higher likelihood) some of the conflict-related sentences of the sentence paradigm (experiment 2) act as such cues. Alternatively, the free associations may be associated with conflicts, impairing subsequent conscious access to them.

It should be pointed out that different from most studies on the effects of emotions and autonomic arousal on memory, our paradigm does not allow one to distinguish unequivocally between an encoding and a retrieval phase. The associations that need to be later remembered are not presented to the participants, but generated by them. Generation of words during free association is related to conceptual implicit memory for these associations. Therefore, both the free association and the subsequent recall phase depend on retrieval, but retrieval occurs implicitly in the free association phase and explicitly in the recall phase. Repression is defined as an inability to consciously access memory, with remaining implicit memory traces that may lead to psychopathological symptoms. This predicts that associations which cannot be retrieved consciously are still accessible via free association.

During the second phase of our paradigm, participants voluntarily and consciously attempted to retrieve this information. Therefore, one would predict impairment in the retrieval of associations which can only be accessed implicitly. Again, it should be pointed out that an inability to access information consciously does not allow one to draw specific inferences on the reasons why these associations cannot be retrieved.

Stress, arousal, and cortisol also exert an effect on memory retrieval: Several studies have demonstrated that high cortisol level and stress significantly impair conscious memory retrieval [51], [52], while the effect on implicit memory recall appears to be reduced or even absent [53], [54]. Based on these findings, one may assume that also conflict-related memories, whose recall putatively causes a relevant amount of stress, may be accessibly in implicit memory tasks, but difficult or impossible to retrieve consciously. On the other hand, such conflict-related situations were already encoded during stressful conditions (which would improve their memory), making it difficult to predict effects on subsequent retrieval.

### Possible Bias 1: Conscious Suppression

We tried as best as we could to encourage participants to associate freely, i.e. to tell the first word which came to their minds. A few days prior to the scanning procedure in the second experiment, we scheduled an appointment with each participant to train this method (in the absence of an experimenter). Furthermore, participants were told that their associations during scanning could not be perceived by the experimenter, but were only recorded for subsequent pseudonomized analysis by a trained psychotherapist.

However, since participants were not in a therapeutic relationship to the experimenter, it is likely that they were inhibited to tell indeed everything which came into their minds. Therefore, the first words which were generated by the participants may not have been the first words which came into their minds, but “censored” versions. In this case, delayed reactions and increased skin conductance responses would indeed be due to a conflict with internalized social norms, but this conflict would not be unconscious, and it would not lead to repression, but to (more or less automatic) response inhibition. An interpretation of our results in light of conscious response inhibition might be supported by the finding that in the second paradigm, consent was lowest for the conflict sentences, and in general, lower consent predicted impaired memory. However, partial correlation analysis could rule out that the relationship between RT (and SCR) and memory was solely due to consent. Future studies may try to address this issue, e.g. by inviting patients undergoing psychoanalysis together with their analysts (as experimenter); in this case, it is likely that responses were given in a less restricted manner (although of course effects of appropriateness cannot be excluded during psychotherapy either).

### Possible Bias 2: Cognitive Factors

It might be argued that forgetting in our paradigms is not related to repression of conflicts, but rather to cognitive factors. For example, the easiness with which an association is found might affect subsequent memory for this association, and could as well be reflected in RTs and SCRs. If a cue word is semantically very closely related to an association, the association would be found very rapidly (short RTs) and with very low effort (low SCRs) and easily remembered. In the previous study by Levinger and Clark [25], the relatedness of cue and association was measured as “response entropy”, which was quantified by the number of different responses given across participants for each word in the group. They found that this measure was highly correlated with retest-reliability for each word, i.e. when they tested participants four weeks later, the probability that the participants gave the same response as during the first recall was significantly higher when relatively few different associations were given to this word during the initial association phase. Moreover, they found that higher response variability predicted forgetting and was positively correlated with RTs and SCRs. Importantly, though, partial correlation analysis showed that the correlation of SCRs and RTs with memory remained significant after partialing out the effects of response entropy. In other words, the correlation of SCR and RT with memory was independent from the correlation of response entropy with memory. We found that the correlations of SCRs and RTs with memory remained qualitatively similar when we controlled for the influence of response entropy. While these analyses argue against the hypothesis that our results are solely due to the difficulty with which an association can be generated, these analyses do not rule out that other measures of semantic relatedness between presented and associated word contribute to subsequent memory. Therefore, we used two measures similar to latent semantic analysis [40], [41] to exclude that memory was due to the semantic similarity between cue word and the freely generated association. An even more direct test would be to assess semantic relatedness in individual subjects by interrogating them of the subjective similarity between presented and associated word.

### Possible Bias 3: Differences during Encoding and Retrieval

Another potential problem with our approach is that the tasks during free association and conscious retrieval attempts were very different, in particular since participants were informed that successfully retrieved words would be rewarded and incorrect intrusions punished (which may introduce effects of reward). Monetary rewards and punishments were provided to ensure that participants attentively tried to retrieve as many words as they could (and that lack of retrieval was not due to insufficient retrieval effort). Therefore, this feature of our paradigm may reduce the number of trials which would have been incorrectly labeled as “repressed” if participants had not tried hard enough to retrieve an association. Moreover, it should be noted that possible effects of reward may only play a role during the comparison of subsequently forgotten and subsequently remembered words in the first experiment, but not during the comparison of associations to conflict, negative and neutral sentences in the second experiment, because retrieval success was rewarded in all conditions equally. However, the differences in internal cues during the free association and the retrieval phase may also impair memory performance (e.g., [35]). Therefore, some associations may not have been remembered because of an inconsistency between initial free association and subsequent recall. Overall, we would like to emphasize that while our current results are consistent with repression theory, a variety of alternative explanations cannot be excluded. Future experiments should address these issues more extensively.

### Summary and Future Directions

To summarize, our two fMRI studies provide consistent evidence that autonomic arousal during free associations predicts subsequent forgetting, that this effect depends on an activation of conflict-related regions such as the adACC and a down-regulation of regions within the medial temporal lobe, which is known to be crucial for episodic memory recall [7]–[9]. This pattern of results fits exactly to the psychodynamic theories of repression as a mechanism for avoiding conscious access to conflict-related material. One relevant future project will be to test the effects of individually-designed stimuli, e.g. derived from psychotherapy or operationalized psychodynamic diagnostics (OPD) [55]. Furthermore, it will be interesting to apply this paradigm to clinical populations whose psychopathology is assumed to depend on repression, for example patients with conversion disorders or dissociative pseudo-seizures. Possibly, brain activation patterns during this paradigm may point towards relevant unresolved conflicts – reminiscent of the initial ideas of C.G. Jung, and in line with previous research in the emerging new field of “Neuro-Psychoanalysis” [56]–[58].

## Supporting Information

Table S1 **Instructions to Experiment 1 (free association phase, retrieval phase, rating phase).**
(DOC)

Table S2 **Instructions to Experiment 2 (free association phase, retrieval phase).**
(DOC)
