# Supplementary material for: Can the Neural Basis of Repression Be Studied in the MRI Scanner? New Insights from Two Free Association Paradigms
Source: PLoS One. 2013 Apr 30;8(4):e62358. doi: 10.1371/journal.pone.0062358 (PMC3640070; doi:10.1371/journal.pone.0062358)
Supplement: Table S1 — Instructions to Experiment 1 (free association phase, retrieval phase, rating phase). (DOC) [file pone.0062358.s001.doc]

Dear participant,

Thank you for participating in our experiment.

In this experiment, we would like to investigate subjects’ reactions to different stimulus words.

In the first part, you will see single words on your video goggles, to each of which you should speak out the first word that comes to your mind *as quickly as possible.*

If, for example, you are shown the word „table“, and the first word that comes to your mind is „chair“, you should immediately say „chair“.

Please try not to exert any conscious control on this procedure.

“Turn off your brain.”

Of course, your answers will be kept strictly confidential!

Right after the experiment, your answers will be assigned a code number, so that during analysis, the identities of the subjects remain unknown.

table

„chair“

Do you have any questions?

*Good luck!*

Dear participant,

The second part of the experiment deals with your memories of the associations generated before.

You will be shown the same words again, in a different order.

For each word that you see, please try to remember the word that came to your mind in the first part of the experiment, and speak it out clearly.

If, for example, you are shown the word „table“ and you remember having said „chair“ in the first part of the experiment, say “chair”.

Please name the word as quickly as possible, if you are sure. You cannot correct yourself. If you don’t remember the word, or if you are not sure, try to guess.

For each correct answer, you will be rewarded with 10ct, for each incorrect or missing answer, you will lose 5ct.

The next word will appear after approximately 12 seconds.

table

„chair“

…remember…

Do you have any questions?

Dear participant,

The last part of the experiment is a rating of the words:

Which words were *for you* positive, which were negative or neutral?

You will have the possibility to assess this on a 9-step scale:

-4 -3 -2 -1 0 1 2 3 4

negative neutral positive

How strong is the feeling accompanying the word, how strongly did you feel aroused or agitated?

Here, too, you can choose from a 9-step scale:

1 2 3 4 5 6 7 8 9

slight inner moderate strong inner

arousal inner arousal arousal

Since the scale has 9 steps, you may well use a broad range for your ratings.

After every rating, the next word will appear.

table

press button

house

press button

….

Do you have any questions?
