# Supplementary material for: Can the Neural Basis of Repression Be Studied in the MRI Scanner? New Insights from Two Free Association Paradigms
Source: PLoS One. 2013 Apr 30;8(4):e62358. doi: 10.1371/journal.pone.0062358 (PMC3640070; doi:10.1371/journal.pone.0062358)
Supplement: Table S2 — Instructions to Experiment 2 (free association phase, retrieval phase). (DOC) [file pone.0062358.s002.doc]

Dear participant,

thank you for participating in our experiment.

In this experiment, you will be shown 24 sentences, and you should speak out the first 3 words that come to your mind as quickly and spontaneously as possible, and then associate freely for approximately a minute.

If, for example, you read the sentence „I sat at the table“, you might think of: „*chair, sit down, carpenter*”. Please say these words out loud. Then, you will start the free association, e.g.: “*Yesterday, I wrote a letter sitting at the table. On the way to the mail box I met Dirk. I once spent a vacation with him in Italy. There, we went swimming a lot. Next week I will go to the swimming pool,* … etc.“

You should try to get into a “flow”, and speak out thoughts right as they come to your mind – without a “brake”.

Please try not to exert any conscious control on this procedure. “Turn off your brain.”

**Of course, all your associations will be kept strictly confidential!**

Neither the leaders of the experiment, nor anybody you will meet during the experiment, are authorized to listen to the free association part of your answers. Right after the experiment, a code number will be assigned to your answers, so that during analysis, the identities of the participants remain unknown.

Since the free associations you generate may be used in an pseudonymized form for research by other investigators from our clinic (but not by anybody you meet during the experiment), please try to speak audibly and clearly.


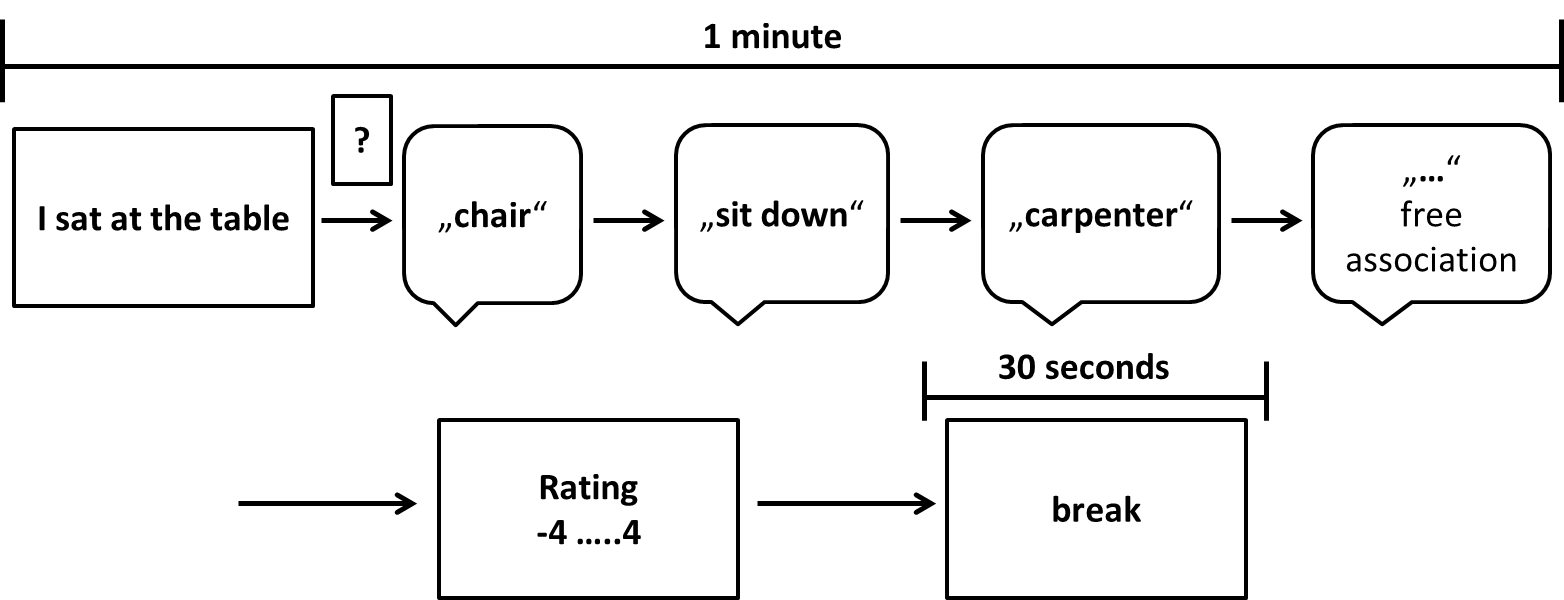


Right after the free association, you will be asked to answer these questions:

How strongly does the sentence apply to you personally?

X ✓

1 2 3 4 5 6 7 8 9

Not at all a little medium rather completely

How do you feel right now? How is your *mood* after this association?

-4 -3 -2 -1 0 1 2 3 4

negative neutral positive

How strongly do you feel *activated* or *calmed* by the sentence to which you associated?

1 2 3 4 5 6 7 8 9

very calmed neither/nor very activated

Do you have any questions?

*Good luck!*

Dear participant,

the second part of the experiment deals with your memories of the associations generated before.

You will be shown the same sentences again, in a different order.

For each sentence, try now to remember the 3 words that came to your mind in the first part of the experiment, and speak it out clearly.

If, for example, you see the sentence „I sat at the table“ and you remember having said “chair”, “sit down”, and “carpenter”, say now “chair, sit down, carpenter”.

You have 30 seconds per sentence to remember. The next sentence will appear automatically. You cannot correct yourself, that means every word you say will be counted as an answer.

For each correct answer, you will be rewarded with 10ct, for each incorrect or missing answer, you will lose 5ct.

„chair“

„sit down“

„carpenter“

I sat at the table

remember…

Do you have any questions?
